# Role of Extracellular Matrix in Development and Cancer Progression

**DOI:** 10.3390/ijms19103028

**Published:** 2018-10-04

**Authors:** Cameron Walker, Elijah Mojares, Armando del Río Hernández

**Affiliations:** Cellular and Molecular Biomechanics Laboratory, Department of Bioengineering, Imperial College London, London SW7 2AZ, UK; c.walker17@imperial.ac.uk (C.W.); e.mojares17@imperial.ac.uk (E.M.)

**Keywords:** tumour microenvironment, cancer progression, extracellular matrix, matrix remodelling, fibrosis

## Abstract

The immense diversity of extracellular matrix (ECM) proteins confers distinct biochemical and biophysical properties that influence cell phenotype. The ECM is highly dynamic as it is constantly deposited, remodelled, and degraded during development until maturity to maintain tissue homeostasis. The ECM’s composition and organization are spatiotemporally regulated to control cell behaviour and differentiation, but dysregulation of ECM dynamics leads to the development of diseases such as cancer. The chemical cues presented by the ECM have been appreciated as key drivers for both development and cancer progression. However, the mechanical forces present due to the ECM have been largely ignored but recently recognized to play critical roles in disease progression and malignant cell behaviour. Here, we review the ways in which biophysical forces of the microenvironment influence biochemical regulation and cell phenotype during key stages of human development and cancer progression.

## 1. Introduction

The extracellular matrix (ECM) is most commonly defined as the non-cellular component of tissue that provides both biochemical and essential structural support for its cellular constituents. Rather than serving simply as an intercellular filling, the ECM is a physiologically active component of living tissue, responsible for cell–cell communication, cell adhesion, and cell proliferation [1]. Fundamentally, the ECM is composed of and interlocking mesh of water, minerals, proteoglycans, and fibrous proteins secreted by resident cells. However, every organ has a unique composition of these elements to serve a particular tissue-specific purpose [1,2]. Indeed, this unique composition arises through dynamic biophysical and biochemical feedback between cellular components and their evolving microenvironment during tissue development [3,4]. For any specific tissue, components of the ECM are created and arranged by resident cells in accordance with the needs of the tissue. The production of essential fibrous proteins, such as collagen, elastin, and laminin are controlled by the ECM and adapt during various stages of embryonic development and disease progression. As a highly dynamic structure, the ECM is constantly undergoing a remodelling process, by which components are degraded and modified, facilitated primarily by ECM proteinases [5,6]. The balance between degradation and secretion of ECM, orchestrated by ECM-modifying cells, is responsible for tensional homeostasis and the properties of each organ, such as elasticity and compressive/tensile strength.

In vitro, most animal cells are known to only maintain viability when adhered to a substrate [7]. In this regard, cells rely heavily on their sense of touch to survive by protruding, adhering, and spatially interacting with the surrounding ECM. Various cellular growth factor receptors and adhesion molecules along the cell membrane, such as integrins, are responsible for the cell’s ability to adhere and communicate with its environment [8,9]. Indeed, cells have been shown to transduce cues from the ECM, such as spatial context and mechanical rigidity, to coordinate crucial morphological organization and signalling events through regulation of gene transcription. This process in which a cell converts external mechanical stimuli into a downstream intracellular chemical signal is known as mechanotransduction [10]. The sensitivity by which cells respond to biophysical and biochemical cues of the ECM demonstrates the importance of tissue homeostasis in the maintenance of healthy resident cells. Accordingly, dysregulation of ECM remodelling has been shown to contribute significantly to cell fate through various fibrotic conditions, characterized by excess ECM deposition and increased rigidity [11]. Due to increased interstitial pressure, unresolved loss of tissue homeostasis has been linked to an elevated risk of various conditions, such as osteoarthritis, cardiovascular disease, and cancer [11]. In this review, we will discuss the role of the ECM in critical physiological processes, such as tissue development and cancer, and some potential targets for therapeutic intervention.

## 2. Primary Components of the Extracellular Matrix (ECM)

The ECM is composed of various proteins that give rise to different structures and properties that exist within it. The main components of the ECM include collagen, proteoglycans, laminin, and fibronectin. Even among these ECM components, there are subtypes that further specify their function in the overall structure and properties of the ECM. As structure dictates function, different subtypes and combinations of ECM molecules confer different functions that are essential for the whole body to function.

### 2.1. Collagen as the Basis of ECM Architecture

Collagen is the most significant component of the ECM and the most abundant protein in human tissue, with 28 unique subtypes discovered [12,13,14,15]. Each type is composed of homotrimers or heterotrimers of left handed helical α chains that are twisted to form a right handed triple helix structure [13,16]. The collagen superfamily is a large group of proteins that contain the Gly-X-Y motif, where X and Y are usually either proline or hydroxyproline [16,17]. Despite the large amounts of bulky proline, the right-hand helical structure is stabilized by the small glycine, interchained hydrogen bonds, and electrostatic interactions involving lysine and aspartate [17,18]. Fibrillar collagens form fibrous structures often found in tendons, cartilage, skin, and cornea [13,14]. Each collagen fibre is made up of several subtypes of collagen in response to its tissue location. The most abundant type of fibrillar collagen, type I collagen, and can be found in connective tissues ranging from skin and bone to tendon and cornea [19]. Collagen I is involved heavily in processes such as a wound repair and organ development.

**Figure 1:**
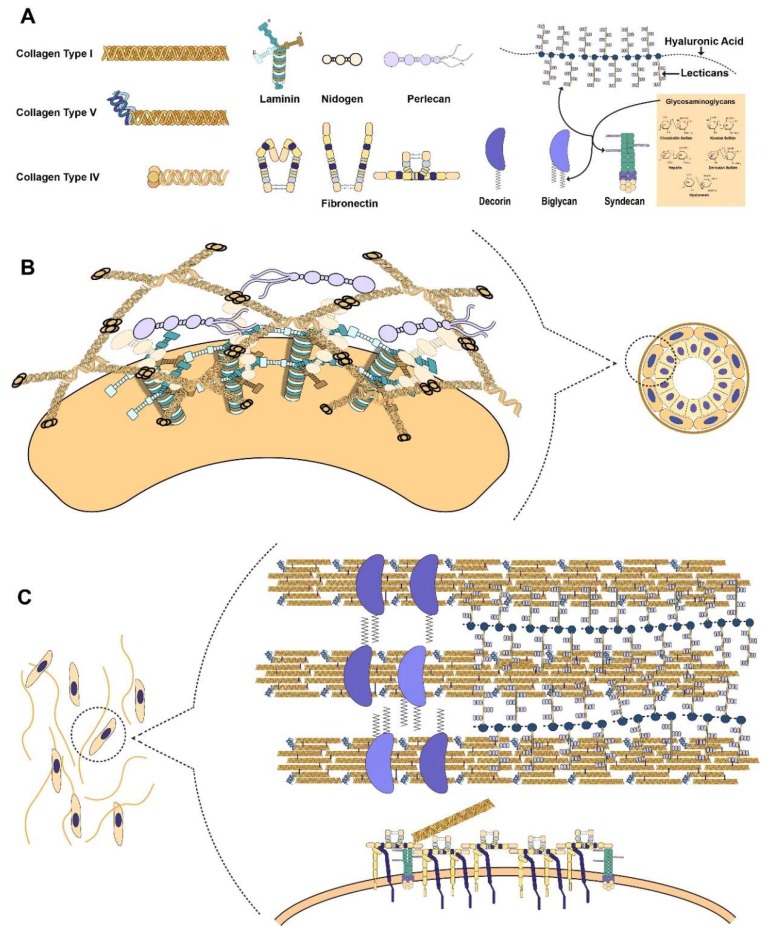
Unique ECM molecules and their organization in the basement membrane and interstitial stroma. Panel **A** (top) shows the unique components of the extracellular matrix. Panel **B** and **C** (middle) shows how these different collagens, proteoglycans, laminins, and fibronectin are organized within the basement membrane (**B**) and interstitial ECM (**C**). A breast acinus with epithelial cells is surrounded by myoepithelial cells and the basement membrane. In the basement membrane, the laminin is bound to the cell and forms a network through its long arms. It is then connected to the collagen IV network through nidogen and proteoglycans such as perlecan and agrin. Outside the basement membrane is the interstitial ECM where fibroblasts that produce and remodel the ECM can be found. In the interstitial stroma, collagen fibres are made up of fibrils composed of collagen I and collagen V. The different proteoglycans, such as decorin, biglycan, and hyalectans, holds the fibrils together to form a collagen fibre. Fibronectin is bound to the cell via integrins and syndecans. Once fibronectin is unfolded, it reveals cryptic binding sites for heparan sulfate proteoglycans (HSPGs) and collagen. Modified and combined figures from Mouw et al. 2014 [15] and Hohenester and Yurchenco 2013 [34].

All fibrillar collagens are first produced as precursors. The α chains are assembled together in the rough endoplasmic reticulum to form the triple helical structure. Proline and lysine are hydroxylated and the molecule is glycosylated to initiate the formation of the triple helical structure [20]. The procollagen is then brought to the Golgi apparatus where it is prepared for cellular export. Processing of the procollagen happens either during or after secretion in the ECM [21,22,23,24]. The C terminal propeptide is cleaved off by specific matrix metalloproteinases (MMPs) and if it is not removed, it leads to high solubility of collagen that prevents it from forming fibrils [25]. For collagen types I, II, and III, the N-propeptides are cleaved off, while for type V, XI, and other fibrillar collagens, the N-propeptides remain (Figure 1A). This modifies the shape and diameter of the fibril without affecting fibril formation [15,25,26,27]. The N-propeptides of type V and XI collagens protrude from the gaps between collagen molecules to prevent lateral growth via steric hindrance and charge interactions [25,26]. Type V and XI collagens are currently believed to be responsible for nucleating and modulating the fibril formation of collagen [25,26]. It has been shown that the deletion of collagen V in mice leads to failure of fibril assembly despite its low amounts in the total collagen content in most tissues [28].

Once the microfibrils are formed, these may bind with other microfibrils so that they will grow into larger fibres. This process is mediated by other ECM proteins (Figure 1C) [29]. Small leucine rich proteoglycans (SLRPs) such as decorin and biglycan have collagen binding motifs allowing them to modulate fibre growth, size, morphology, and content [15,29,30]. Another subfamily of collagen are fibril-associated collagens with interrupted helices (FACIT) that do not form fibrils themselves but are associated with the surface of collagen microfibrils [13]. Their primary function is to mediate the formation of a higher-order structure via binding with other extracellular matrix proteins such as SLRPs and proteoglycans [26,31]. The supramolecular assembly of collagen is further stabilized by lysyl oxidase (LOX), which leads to overall enhanced mechanical properties. The N terminal and C terminal ends of individual collagen molecules are covalently cross linked by LOX both within and between microfibres, contributing to the great tensile strength of collagen [31,32].

In addition to fibrillar and FACIT collagens, there also exist network forming collagens such as type IV, VIII, and X. These are found in the basal lamina of basement membranes (Figure 1B) [13]. Collagen IV forms a tetramer through their 7S N-terminal domain. Each of these collagen IV molecules is bound to another collagen IV molecule via their C-terminal NC1 domain of each α-chain, forming a hexamer [13]. These two domains of collagen IV allow it to form a stable collagen network that separates the basal lamina from the interstitial stroma [33]. Other ECM proteins such as laminin, nidogen, and perlecan can be found in the basal lamina that strengthens this barrier to effectively maintain the organization of the cells in the body (Figure 1B) [33,34].

Although different types of collagen are able to build various types of supramolecular structures that form the basis of the architecture of the ECM, the contribution of other ECM proteins such as proteoglycans, laminins, and fibronectin cannot be ignored. They largely influence the chemical and physical properties of the extracellular matrix such as through their growth factor binding motifs and innate chemical properties. Furthermore, they also serve as connectors between the cells and the ECM.

### 2.2. Proteoglycans as Functional Modifiers of the ECM

Proteoglycans are characterized as proteins that have glycosaminoglycans (GAGs) covalently bonded to them. These GAGs are long chains of negatively charged disaccharide repeats that can either be heparin sulphate, chondroitin/dermatan sulphate, hyaluronan, or keratin sulphate. Due to the negative charge of these GAGs, proteoglycans are able to sequester water and cations, which gives them their space-filling and lubrication functions [35]. For the purpose of this review, only transmembrane proteoglycans and those found in the pericellular and extracellular space will be discussed.

Of the 13 transmembrane proteoglycans, four of them are syndecans, proteins thought to act as co-receptors [35]. Syndecans have an intracellular domain, transmembrane domain, and ectodomain (Figure 1A). The GAGs, typically heparan sulphates, are found attached to the ectodomain, which can be shed through the action of MMPs [35,36]. The ectodomain of syndecans is intrinsically disordered, which allows it to interact with a wide variety of molecules to perform a broad range of biological functions (Box 1) [35]. Some of its functions involve binding to growth factors and morphogens, facilitating exosome uptake, and being co-receptors of receptor tyrosine kinases [36,37,38,39].

One of the proteoglycans found in the pericellular area of the basement membrane is perlecan. As a large heparan sulfate proteoglycan (HSPG), perlecan has multiple domains, each with different binding sites and functions (Figure 1A) [40]. These heparan sulfates can bind to a variety of molecules such as growth factors, growth factor receptors, collagen, and other ECM proteins. In the basement membrane, perlecan binds and links collagen IV, nidogen, and laminin in order to further strengthen the basement lamina (Figure 1B) [33,34,41].

Proteoglycans found in the extracellular space are classified into hyalectans and SLRPs. The structure of hyalectans are identical: the hyaluronic acid binding N terminal and lectin binding C terminal with GAGs are attached between the N and C terminal ends (Figure 1A). Hyalectans are encoded by 4 distinct genes: aggrecan, versican, neurocan, and brevican [35]. Aggrecan is found mostly in bone cartilage and the brain while neurocan and brevican are found in the central nervous system. On the other hand, versican is found in the ECM of almost all tissues and organs [42]. They can serve as molecular bridges between the cell surface and the extracellular matrix [35]. Versican has been shown to bind to collagen type I and fibronectin, which are both substrates of integrins [43]. The binding of versican to fibronectin’s RGD motif leads to loss of cell adhesion as it sequesters fibronectin from the cell’s integrins [42,43].

SLRPs make up the largest family of proteoglycans due to its 18 distinct gene products each with multiple splice variants and processed forms [35]. These proteins have a relatively short protein core with a central region dominated by leucine-rich repeats (LRRs). They are expressed in the ECM during development of various tissue types, suggesting their critical involvement in directing organ size and shape during embryonic development and homeostasis [44,45]. Decorin and biglycan are SLRPs that have collagen-binding motifs and regulate collagen fibre assembly along with other proteoglycans (Figure 1C) [46].

In summary, proteoglycans vary in form and structure that confer different functions in the ECM. They are integral in the maintenance of a healthy ECM without which would lead to a non-functional ECM and a collapse of its structure.

Box 1 **Sensing the extracellular matrix’s (ECM) mechanical properties.**

The ECM is sensed by the cell through transmembrane proteins such as integrins and syndecans and other glycoproteins. Integrins are one of the most versatile transmembrane proteins as various heterodimer combinations allows it to bind to fibronectin, laminin, and collagen [47]. Integrins themselves are mechanosensors. Stretching has been shown to increase integrin binding to the ECM via conversion of integrins to its high-affinity state in smooth muscle cells and fibroblasts [48]. Integrins also experience a conformational change in their cytoplasmic domains, allowing it to activate several signalling pathways such as mitogen-activated protein (MAP) kinases and Rho GTPases [49,50,51]. Syndecans can also bind to fibronectin, resulting in a synergy between integrin and syndecan to activate signalling cascades through focal adhesion kinase (FAK) and subsequent focal adhesion complex stabilization [52]. There are other receptors for other ECM components such as CD44 for hyaluronan, 67 kDa laminin receptor for laminin, and discoidin domain receptors (DDRs) for collagen [53,54,55,56,57].

Integrins and syndecans activate various pathways such as the MAPK and Rac1/RhoA pathways. The selective activation of these pathways leads to context-dependent regulation of cell survival, growth, proliferation, motility, spreading, or migration [52,58]. Integrins are connected to the actin cytoskeleton through vinculins, talins, and other scaffold proteins while syndecans are connected to the microfilaments through syntenin and through the actin cytoskeleton via a-actinin [52,58,59,60,61]. The adhesion complexes formed by integrins and syndecans have been found to be mechanosensitive [9,62]. The intracellular signalling and mechanotransduction through these receptors is still an active field of research. Much is still to be discovered about the pathways that facilitate ECM mediated cellular responses.

### 2.3. Connecting the Cell to the ECM through Laminin

Laminins are trimeric glycoproteins consisting of α, β, and γ chains that are often found in the basal lamina or some mesenchymal compartments [15]. The 12 mammalian α, β, and γ chains can theoretically create 60 unique laminins but only 16 combinations have been observed so far [34,63]. The α chains vary in size from 200 and 400 kDa while β and γ chains have sizes from 120 to 200 kDa. A trimer can then have a size varying from 400 to 800 kDa [63]. During rotary shadowing electron microscopy, laminins look like cross-shaped molecules [34,64,65]. The three chains form an α-helical coiled coil structure that forms the long arm of the cross while the three short arms are composed of one chain each (Figure 1A) [34]. At the end of the long arm are 5 laminin G-like (LG) domains from the α chain that serve as attachment sites for the cell. Integrins, dystroglycan, Lutheran glycoprotein, or sulfated glycolipids bind to these LG domains [63]. At the end of each short arm are laminin N-terminal (LN) domains that are important for laminin polymerization and basement membrane assembly (Figure 1B) [34].

Laminins have cell type-specific functions such as adhesion, differentiation, migration, phenotype maintenance, and apoptotic resistance [63]. Through binding of integrins, laminins are able to create a dynamic link between the cell and the ECM (Box 1) [63]. Unique heterotrimeric laminins will have unique integrin heterodimers binding partners to allow the induction of signalling pathways and organization of intracellular cytoskeleton [63,66]. Collagen IV deposition in the basement membrane is seen as the maturation of the basement membrane that is essential for structural stability later in development [34,67]. However, the exact mechanism by which laminins bind to collagen IV remains unclear. Initial studies indicated that nidogen binds to laminin through the LE domains of the γ1 chain and collagen IV, thus serving as an intermediary between the two networks found in the basement membrane. However, recent research has indicated that nidogen might not be the major bridge in connecting laminins and collagen IV [34]. It has been observed that the interaction between laminins and collagen IV is directly mediated by heparan sulfates [68]. Perlecan was thought to mediate this function, but genetic ablation of perlecan in mice did not result in collagen IV depletion [34,67]. It has since been postulated that agrin, another pericellular HSPG, serves as a compensating candidate. In this model, both perlecan and agrin would bind to the nidogen containing laminin network and to the collagen IV’s 7S and NC1 domains (Figure 1B) [69,70]. Laminins serve crucial roles in both basement membrane assembly and ECM–cell interactions. Recent studies have indicated that basement membrane assembly is initialized through laminin polymerization [53,54,55,56,57,58]. Indeed, genetic ablation of either β1 or γ1 chains proved to be lethal due to the resultant failure of basement membrane assembly. While collagen, proteoglycans, and hyaluronic acid comprise the major structural component of the ECM, laminins are one of the molecules that bridge the interaction gap between the cells and the ECM [15].

### 2.4. Fibronectin as the Mechanosensitive Connection Between the Cell and ECM

Fibronectin is a multi-domain protein that interacts with the various previously described ECM components to connect the cell to the ECM [15]. It is encoded by a single gene, but it has 20 isoforms in humans as a result of alternative splicing of the mRNA [71,72]. Similar to collagen, fibronectin forms a fibrillar network in the ECM (Figure 1C) [71]. Fibronectin naturally exists as a dimer outside the cell, mediated by the two cysteine disulfide bonds, which is crucial for its ability to assemble in a fibrillar fashion (Figure 1A) [71,73]. Fibronectin matrix assembly is mediated by selective binding to α5β1 integrins through an RGD binding motif and a synergy site on the fibronectin molecule [59,62]. Through these integrins, the compact and soluble secreted fibronectin is unfolded revealing cryptic binding sites for other fibronectin molecules to form the fibronectin fibrillar network (Figure 1C) [1,71,74]. Anti-integrin and anti-fibronectin antibodies have been shown to prevent fibronectin fibril formation [71,75,76]. Fibronectin binding induces integrin clustering that provides local high concentrations of fibronectin at the cell surface. This phenomenon promotes fibronectin–fibronectin interactions through the N terminal assembly domains of each molecule [71].

Once fibronectin is tethered to the cell surface by integrins, the actin cytoskeleton can pull onto fibronectin molecules to change its conformation [71,72]. This will affect the C terminal regions of fibronectin, revealing cryptic binding sites for fibronectin, heparan sulfates, heparin, collagen, and other ECM proteins [77,78,79,80,81]. It is through strong non-covalent protein-protein interactions that the fibronectin network matures and becomes insoluble, although other ECM proteins may mediate mature lateral interactions between fibrils [71]. These interactions stabilize the relatively weak binding sites at individual sites. However, the turnover of the fibronectin matrix is still largely unexplored [71].

Due to fibronectin’s multiple binding sites for other ECM proteins, it has been implicated in various functions, including a role in collagen type I assembly. It has been shown that in the absence of fibronectin, collagen fibrils do not accumulate, suggesting a role for fibronectin in collagen assembly [82,83]. However, this relationship may prove reciprocal as recent studies have also implicated that collagen has a role in enhancing fibronectin assembly [71,84,85].

## 3. Function of ECM

**Figure 2:**
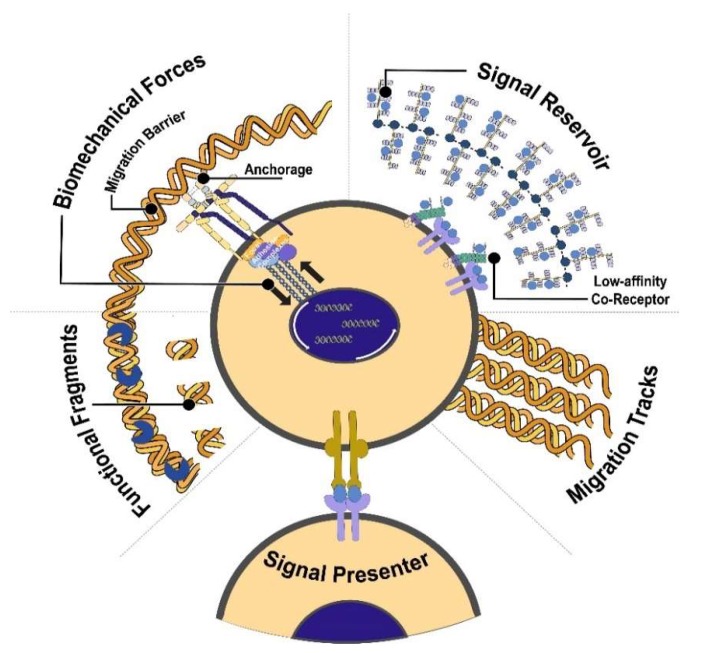
Functions of the ECM. The ECM serves as a point of anchorage for the cells that is essential for maintaining tissue polarity and asymmetric stem cell division. Depending on the context, it can impede or facilitate migration. It can sequester growth factors and prevent its free diffusion. Other ECM components can bind growth factors and can serve as co-receptors or signal presenters, which help determine the direction of cell-cell communication. Through the action of metalloproteinases (MMPs), fragments of the ECM can also influence cell behaviour. The physical properties of the ECM can be sensed by focal adhesion complexes, which lead to a variety of changes in cell phenotype such as reorganization of the 3D genome. Figure modified and adopted from Lu, Weaver, and Werb 2012 [90].

The plethora of unique ECM molecules serves several functions that influence biochemical and biophysical processes in the cell simultaneously (Figure 2). While the ECM has been considered for many years as an inert scaffold solely providing structure for the cells, its role in determining the functions and phenotypes of cells has clearly emerged in the last two decades. The ECM can serve as binding sites, controlling the adhesion and movement of cells [86]. This is emphasized in the complex structure and composition of the basement membrane that serves as a barrier between epithelial cells and the interstitial stroma [6,87]. In addition to structural integrity and anchorage, the ECM components have several binding sites for growth factors, controlling their release and presentation to target cells. This is especially important in morphogenesis as it establishes morphogen gradients [88]. Finally, the ECM transmits mechanical signals to the cells, which activates several intracellular signalling pathways and cytoskeletal machinery [89]. Indeed, the ECM serves several functions and here we review the function of the ECM in the context of development and maintenance of the stem cell niche.

The function of the ECM is best described in the context of development. The development of a mammalian embryo from a foetus to a fully developed organism is a well-orchestrated phenomenon that involves carefully controlled mechanisms. In such a relatively rapid process, the spatiotemporal composition, amount, and characteristics of the ECM must be tightly regulated. Several studies have shown that mutated ECM components lead to birth defects or embryonic lethality, which emphasizes its role in development [2,87]. The geometry, rigidity, and other physical properties of the ECM are sensed by the cells and ultimately direct their differentiation and the complex spatial and structural arrangements they form in tissues (Box 1).

### 3.1. ECM as Tracks for Migration and Proliferation

Migration of cells is essential for tissue development and can be best illustrated by neural crest cells, which migrate from the periphery of the neural tube to different parts of the embryo to form parts of the heart, spinal nerve, skin, and cranium [91]. How these cells direct their migration and final destination is a complex question that has been extensively studied.

The ECM influences the migration track and speed of migrating cells through its topography, composition, and physical properties. The alignment of the underlying ECM has been shown to direct cell migration and proliferation. Sharma et al. [92] previously used aligned fibres to direct cell migration in the context of wound healing in vitro. Moreover, self-aligning materials have been recently used to create ECM constructs for brain tissue regeneration in vivo [93].

Cells migrate from regions with lower ECM concentration to higher ECM concentration due to an adhesion gradient in a type of cell migration known as haptotaxis [87]. However, this relationship is nuanced. If the concentration of the ECM is too high, the adhesion force experienced by the cell is too large from them to continue to migrate. Accordingly, the speed of migration is dependent on the ECM concentration as well. As migration is a coordinated interplay between adhesion and deadhesion of the cell onto the ECM, the speed of migration is characterized by a bell-shape function with respect to ECM concentration [94].

The speed of migration is also influenced by the composition of the ECM. Hartman et al. [95] have shown that fibroblasts cultured in rigidity gradients composed of fibronectin exhibited cell migration while those cultured on matrices covered with either laminin or a fibronectin/laminin mixture did not exhibit any migration. These results indicate that the ECM not only serves as a track for migration, but also dictates cell migration due to its mechanical properties. It was previously demonstrated by Wang et al. [96] that matrix stiffness and cell contractility also control RNA localization of genes responsible for cellular organization and signalling to cellular protrusions. Proteases that degrade the ECM also facilitate migration of cells through a process involving the interplay of MMPs, adamlysins, meprins, metalloproteinase inhibitors (MMPIs), and other enzymes [2]. It is important to note that constant ECM remodelling is occurring in development. The ECM components and their concentration are continuously modified to dictate the developmental program. 

### 3.2. ECM as the Dynamic Blueprint for Development

The role of ECM in structural organization is best studied in branching morphogenesis, which involves epithelial buds and tubes invading the surrounding mesenchyme rich ECM [87]. This process can be seen in various parts of the body, including mammary glands, salivary glands, and kidneys [87]. Structural organization emphasizes the key functions played by the ECM and different ECM components such as glycosaminoglycans (GAGs), collagen, and proteoglycans. Furthermore, the ECM is constantly changing as this process occurs, highlighting the spatiotemporal control needed to facilitate the development of these organs.

Branching in the mammary gland occurs at the terminal end buds that have a thin hyaluronic acid-rich ECM and an accumulation of thick ECM composed of collagen IV, laminins 1 and 5, and HSPGs at the flanks [97,98]. Thick ECM provides a structure to maintain the tubular organization by serving as anchors for the cells while the reduced ECM at the end bud facilitates the migration of epithelial cells, specifically the cell-budding process [87,99,100]. However, fibrillar collagen does not only serve as a physical barrier. As it binds to discoidin domain receptors (DDRs) expressed by mammary gland cells, it prevents hyper-proliferation possibly to maintain the tubular structure [87,101].

The architecture of the ECM serves as a guide to control how branching occurs through local anisotropies in terms of tension as well [99]. Topographical variation in structure and elasticity of the ECM provides a blueprint for where cells can bend, twist, and break off to form complex morphologies that are essential for the different organs. Using micro-patterned organotypic cultures, Nelson et al. [102] showed that tissue geometry dictates the position of the branches. Furthermore, Gjorevski and Nelson [103] have shown that endogenous patterns of mechanical stress in the surrounding ECM specify the branching pattern. There are several techniques to study ECM architecture in 3D such as atomic force microscopy (AFM) combined with second-harmonic generation (SHG) [104]. Robinson et al. used image analyses algorithms to analyse AFM and SHG micrographs to monitor and analyse ECM remodelling of pancreatic stellate cells. This technique could similarly be used to study how the mechanical forces and ECM architecture continuously evolve during development.

As the end bud of the mammary gland grows, it continuously degrades the ECM, which in turn releases factors dictating the branching direction of the growing buds [2]. ECM degradation releases collagen fragments tumstatin and endostatin that regulate the migration, survival, and proliferation of these cells [105]. Furthermore, the ECM’s binding sites for morphogens and growth factors, such as Wnt glycoproteins, epidermal growth factors (EGFs) and fibroblast growth factors (FGFs), allow them to sequester and control the release of these factors [35,106]. Through this, the ECM facilitates the formation of a morphogen gradient, which is required for diverse types of cells and structures to develop [88]. The growth of the bud is finally terminated through the deposition of inelastic ECM composed of sulphated GAGs (SGAGs) and collagen I [99].

Overall, the ECM modulates the growth of tissues to form complex structures that are required for these organs to function. The ECM provides structural organization not only through its action as a physical barrier to growing cells, but also by activating intracellular signalling in a time and context dependent manner. The ECM does this through growth factor distribution modulation, physical anisotropies, and anchorage.

### 3.3. ECM as the Driver for Cell Fate

The ECM influences cell fate through the previously discussed morphogenetic gradient in development. However, this is not the only mechanism by which ECM affects cell fate, as the physical properties of the ECM also play a critical role.

The role of ECM composition on cell fate is exemplified in mammary gland differentiation. Even with hormonal stimulation in vitro, mammary gland cells do not secrete milk proteins. However, upon exposure of these cells to laminin-1, they begin secreting milk proteins [107]. This activation is due to integrin binding by laminin-1 leading to phosphorylation of the prolactin receptor, an upstream regulator of STAT5. STAT5 then activates the transcription of milk proteins β-lactoglobulin and β-casein, indicating that an appropriate 3-D ECM microenvironment is critical for cells to function properly [102,107].

Using a simple yet elegant approach, Engler et al. [10] demonstrated that when mesenchymal stem cells (MSCs) are cultured on collagen matrices with various elasticities, the MSCs differentiated into osteocytes, myocytes, and neurons on the substrates that resembled their respective native tissue. The process by which these MSCs are able to sense the elasticity and stiffness mechanical environment before initiating an intracellular signalling cascade to dictate cell fate is known as mechanotransduction. This phenomenon allows the cells to sense the physical properties of the underlying matrix and activate appropriate intracellular pathways [89]. When myosin II, a key molecule in various mechanotransduction-signalling pathways, was blocked, MSCs became insensitive to the matrix elasticity mediated cell differentiation [10]. This study emphasized that the ECM’s physical properties themselves have differentiating capability.

Engler’s results indicate that environmental cues can be relayed to the nucleus biochemically or biophysically [89]. Indeed, often biochemical and biophysical relays work in conjunction with one another to transmit environmental information [89,108]. One prominent example of a mechanosensitive genetic regulator is the pair of the transcription co-activators, yes-associated protein (YAP) and transcriptional coactivator with PDZ-binding motif (TAZ). These functionally redundant transcriptional coactivators are known to play crucial roles in critical cellular processes, such as proliferation, wound healing, fibrosis, and other physiological processes that involve changes in biomechanical properties [109]. Importantly, these transcription co-activators are known to be sensitive to both biochemical and biophysical cues. The nuclear localization of YAP/TAZ is regulated by cell shape, stiffness, ECM topology, and shear stress [109,110,111,112,113,114,115]. The integrin complexes that sense this matrix stiffness are linked to the cytoskeleton, which in turn is linked to the nucleus through the linker of nucleoskeleton and cytoskeleton (LINC) complex, which is composed of nesprins, sun, and lamin proteins [116,117]. This allows direct transmission of mechanical cues from the extracellular matrix to the nucleus [117]. The extensive role of YAP/TAZ in cell processes elucidates the role of mechanotransduction in development and diseases, such as cancer [108].

In addition to matrix stiffness, the response of the cell to the ECM’s physical properties has also been shown to be dependent on the ligand tether length. It has been shown that the focal adhesion sizes and cell-adhesion strengths were affected when the tether length of the ECM coating’s ligand was varied [118]. Coating stiff ECM with RGD ligands with longer tether lengths lead to the cell sensing a softer ECM thereby controlling mechanotransduction mediated YAP/TAZ nuclear localization in cancer. This might be a novel effective treatment against cancer.

## 4. Tissue Homeostasis

The ECM is a highly dynamic structure. Even after development, ECM is constantly being deposited, degraded and modified to maintain tissue homeostasis. This is especially important in maintaining the phenotype of cells and in physiological processes such as wound healing, angiogenesis, and bone remodelling [6,119,120].

To maintain tissue homeostasis, the cells in contact with the ECM sense the properties of the ECM through receptors and focal adhesion complexes. In turn, the cell regulates the expression of ECM components and enzymes based on the signals of the ECM. This creates a feedback mechanism wherein the cell also influences the ECM, which results in a balance of deposition and degradation of ECM components [116].

The response of the cells to other stimuli, including shear stresses exerted by blood flow, are ultimately influenced by the ECM component. Chen and Tzima [121] showed that platelet endothelial cell adhesion molecule-1 (PECAM-1), a mechanosensitive molecule, is essential for vascular remodelling, which occurs in response to long-term changes in hemodynamic conditions. Furthermore, Collins et al. [122] recently demonstrated that the ability of platelet endothelial cell adhesion molecule-1 (PECAM-1) to respond to mechanical forces is influenced by the type of ECM they are adhered to. This exemplifies the complexity and importance of the feedback mechanism that exists between the ECM and the cell to maintain tissue homeostasis.

The importance of the ECM in maintaining tissue homeostasis is exemplified by the study performed by Weaver et al. [123] where they were able to revert the malignant breast cancer cell phenotype to the normal phenotype. They did this by culturing breast cancer cells onto basement membrane based 3-D substrates coated in integrin β1 blocking antibodies [123]. This study confirmed that the ECM is able to override the mutations causing the cancer phenotype, emphasizing the ECM’s role in maintaining the correct cell phenotype. The role of dysregulated ECM in cancer progression will be discussed further in the next section.

That an imbalance in the deposition and degradation of the ECM leads to diseases is a hallmark not just of cancer but also other prominent diseases including fibrosis [124,125,126]. Overall, the ECM’s role in tissue homeostasis is to direct proper cell response and phenotype to maintain the tissue’s mechanical integrity and function.

## 5. ECM in Cancer

### 5.1. Dysregulation of ECM Molecules in Cancer Progression

Traditional perspectives of cancer have shifted to reflect the important role of the ECM in regulating cell proliferation, migration, and apoptosis. On a microscopic level, the particular arrangement and orientation of ECM constituents form a tissue-specific microenvironment that plays a critical role in tumour progression [11,127,128]. It is now understood that the ECM not only undergoes continuous active remodelling, but also elicits biochemical and biophysical cues to influence cell adhesion and migration [129]. As such, small changes in microenvironment homeostasis can have significant effects on the proliferation of cancer cells. As the most significant ECM component, collagen dictates the primary functional properties of the matrix. Indeed, changes in the deposition or degradation of collagen can lead to the loss of ECM homeostasis [130,131].

**Figure 3:**
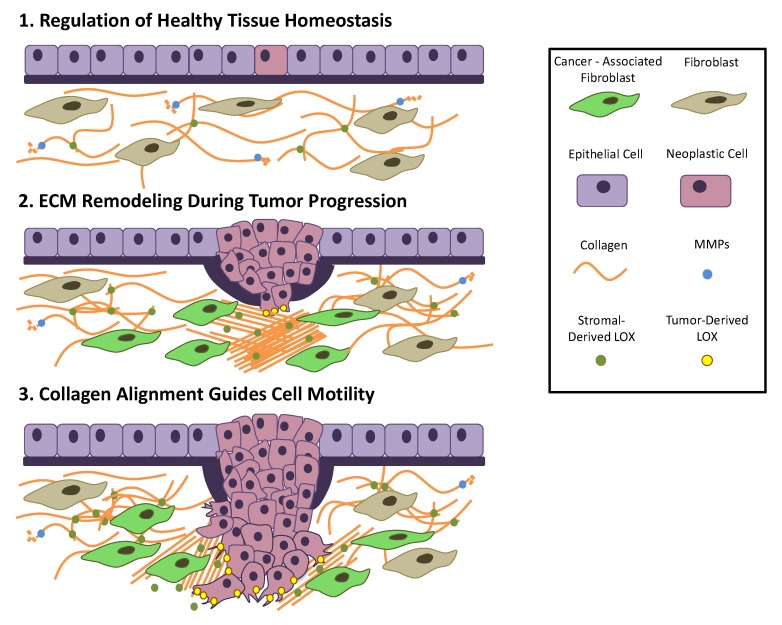
ECM remodelling during cancer progression and initiation. (**1**) Epithelial neoplastic cells proliferate rapidly, inducing strain on the basement membrane. (**2**) Basement membrane bulges due to mechanical strain. Adjacent cancer-associated fibroblasts increase deposition of collagen. Stromal-derived lysyl oxidase (LOX) aligns collagen. (**3**) Neoplastic cells breach membrane and migrate along aligned collagen. (Adapted from Lu et al. [6].)

As tumour cells proliferate, the surrounding ECM undergoes significant architectural changes in a dynamic interplay between the microenvironment and resident cells. These changes, including increased secretion of fibronectin and collagens I, III, and IV illustrate that tumour progression demand a continuous interaction between the ECM and tumour cells (Figure 3) [132]. Increased deposition of matrix proteins promotes tumour progression by interfering with cell–cell adhesion, cell polarity, and ultimately amplifying growth factor signalling [133]. However, the exact role of collagen deposition in tumour progression is nuanced. Recent studies have shown that increased collagen cross-linking and deposition leads to tumour progression via increased integrin signalling [134,135]. Interestingly, however, depletion of fibrillar collagens I and III can also promote malignant behaviour, indicating that biomechanical forces produced by collagen deposition can have both beneficial and deleterious effects on tumour progression [136,137].

Collagen cross-linking can occur in both an enzyme-mediated and non-enzyme-mediated fashion. Regulated collagen cross-linking is coordinated primarily by LOX and the LOX family of amine oxidase enzymes [1]. LOX, secreted by primary tumour cells, is responsible for catalysing the cross-linking of both collagen and elastin, which in turn increases matrix stiffness and total adjacent ECM volume. Increased ECM stiffness activates integrins and augments Rho-generated cytoskeletal tension to promote focal adhesion formation and cell motility [138]. Elevated LOX activity has been clinically associated with increased collagen cross-linking, fibrosis, and elevated risk of cancer metastasis [139]. Moreover, elevated LOX activity found on invasive edges of tumours has been noted to drive actin polymerization, cell contractility, and migration, providing a pathway for successive tumour cells to follow [130].

Visualization of surrounding epithelial tissue during tumour metastasis has revealed localized matrix organization and alignment along the leading edge of invasive tumours [131,140]. Indeed, local cell invasion of these tumours has been observed to be oriented along aligned collagen fibres, suggesting that the linearization of collagen fibres facilitates tumour invasion [141]. It is believed that these densely aligned collagen fibres act as tracks for proliferating neoplastic cells to migrate out of the tumour. Breast cancer serves as an important example of collagen alignment during tumour metastasis. Although collagen within epithelial structures is typically tangled and disorganized, collagenous tissue surrounding mammary tumours is frequently thickened, stiffened, and aligned perpendicularly to the tumour boundary [142]. Recent studies indicate that the topography of matrix fibres increases the efficiency of tumour migration by reducing the protrusions along the collagen fibre, and hence the distance travelled by the migrating cell [143].

Much like collagen and LOX, elevated levels of the glycosaminoglycan hyaluronic acid in the ECM correlates to increased likelihood of malignancy and poor prognosis [144]. As a naturally occurring omnipresent linear polysaccharide, hyaluronic acid is critical in determining the compressive properties of most biological tissues. The combination of tensile resistance due to collagen and compression compliance due to hyaluronic acid creates the ideal biophysical properties for tissue homeostasis [145]. In addition, it has been found that hyaluronic acid is both an induction signal for mesenchymal transition and a migration substrate [146]. Accordingly, hyaluronic acid is frequently used as a biomarker for prostate and breast cancer. While augmented levels of collagen and LOX directly promote ECM stiffness and mechanically drive cell motility and proliferation, the exact role of hyaluronic acid in cancer metastasis remains unclear. However, its dysregulation can serve as a key biomarker for metastasis and cancer invasion.

### 5.2. Protein Unfolding Mediates Mechanotransduction

ECM signalling is a crucial cellular process that drives cell proliferation, differentiation, and defers apoptosis [147]. In brief, if a cell cannot sense its mechanical environment, it cannot survive. Many studies have reported that cells are capable of sensing their microenvironment through chemical signalling, such as growth factors and metabolic precursors [148,149,150]. In order to detect ECM rigidity, it is believed that cells mechanically probe their microenvironment via lamellipodia and sense the mechanical feedback and resistance of their environment through integrin-based focal adhesions, triggering an intracellular signalling cascade [151]. The ability for cells to probe their microenvironment is attributed to the actin cytoskeleton, as inhibition of F-actin polymerization limits the ability of cells to generate force, which induces a biological effect similar to plating cells on a soft substrate. Specifically, the ability for cells to produce internal forces is derived from contractile actin bundles and their upstream regulators, such as Rho-associated protein kinase (ROCK), which are necessary to mechanically sense their environment [109]. While mechanical rigidity clearly has profound effects on cell behaviour, the mechanism that translates mechanical force into gene transcription is not fully understood.

Our recent work has illustrated the importance of protein unfolding in the transduction of mechanical force exerted by the ECM. Indeed, talin, a prominent molecule in focal adhesion complexes that couples focal adhesions to the actin cytoskeleton, has been shown to mechanically unfold during force transmission [152]. Deleted in liver cancer 1 (DLC1) is a negative regulator of RhoA and cell contractility that regulates cell behaviour when concentrated to focal-adhesion complexes bound to talin [153]. Mechanical clamping of the R8 domain of talin prevented mechanical unfolding of the molecule, interrupting downstream signalling of DLC1 and, consequently, cell behaviour [153]. Moreover, single molecule force microscopy revealed that every talin rod subdomain is susceptible to unfolding over a physiologically relevant range of forces between 10 and 40 pN [152]. Because the observed range of talin subdomain stabilities within the focal adhesion complex depend on small structural differences, it is possible the mechanical stability of talin rod bundles could be influenced by a few single point mutations. These mutations could lead to misinterpretation of ECM signals, altering cellular response. Incorrect interpretations of ECM information could influence the behaviour of cancer cells in the tumour microenvironment, potentially triggering DLC1 deactivation, increased cell contractility, and cell migration [152,153].

### 5.3. YAP & TAZ Mechanotransduction in Cancer Progression

As robust regulators of cell proliferation and survival, YAP and TAZ play critical roles in regulating organ development, cell differentiation, and progenitor cell self-renewal [109]. During these processes, the YAP/TAZ proteins actively shuttle between the nucleus and the cytoplasm. While in the cytoplasm, the YAP/TAZ proteins play a relatively passive role, regulating specific signalling cascades, such as the Wnt signalling pathway. Meanwhile, when in the nucleus, they readily interact with DNA-binding transcription factors, particularly TEA domain family members (TEAD), to regulate genetic expression associated with proliferation, a key hallmark of cancer [154,155]. Upon biochemical inhibition, YAP/TAZ accumulate in the cytoplasm, suggesting that the main functionality of YAP/TAZ is gene transcription regulation in the nucleus. Importantly, upon cell detachment from a substrate, YAP/TAZ activity is inhibited; suggesting that YAP/TAZ translocation to the nucleus can be regulated by the F-actin cytoskeleton and mechanical force [156]. Moreover, in mammalian systems, matrix elasticity and cell-spreading geometry are noted to heavily regulate YAP/TAZ nuclear transport and their corresponding physiological processes [157]. Taken together, these results suggest that focal adhesion and cytoskeleton-mediated cell signalling of mechanical rigidity is coupled to the YAP/TAZ pathway to induce metastasis and tumour invasion, indicating a direct chemical pathway linking mechanical force with malignant cellular behaviour.

Although cytoskeletal tension is sufficient for YAP/TAZ nuclear translocation, there exist multiple potential pathways and proteins that mediate YAP/TAZ nuclear translocation. For example, the heparan sulfate proteoglycan, agrin, is most commonly known for its role in the formation of neuromuscular junctions during embryogenesis. However, recent advances in the field have suggested that agrin may also serve as an ECM sensor that stabilizes focal adhesions and facilitates YAP/TAZ nuclear translocation through the lipoprotein-related receptor-4 (Lrp4) and muscle-specific kinase (MuSK) pathway [158,159]. Activation of Lrp4 and MuSK by agrin inhibits the Hippo tumour suppressor pathway, ultimately leading to elevated YAP/TAZ nuclear translocation [158,160]. Agrin depletion was shown to promote the inhibitory phosphorylation of YAP, which forced nuclear YAP to remain in the cytosol [161]. Contrarily, supplementary introduction of agrin into cells cultured on compliant matrices was sufficient for YAP activation [161]. Multiple junctional proteins, including the Angiomotin (AMOT) family of proteins regulate YAP/TAZ in combination with changes in actomyosin contractility [161,162]. AMOT proteins have been shown to directly bind to YAP, inhibiting its function. F-actin competitively binds with AMOT to disrupt YAP:AMOT complexes, releasing YAP from its inhibitory state to translocate into the nucleus [161]. Interestingly, agrin depletion elevated YAP:AMOT binding, which ultimately led to decreased YAP activity [159]. Moreover, recent work demonstrates that the Ras-related GTPase, Rap2, is also a key intracellular mediator that transduces ECM rigidity signals to influence YAP/TAZ nuclear translocation [163,164]. At low ECM stiffness, Rap2 is known to bind and activate MAP4K4, MAP4K6, MAP4K7, and ARHGAP29, which stimulate LATS1a and LATS2 while inhibiting YAP and TAZ nuclear translocation [163]. These findings demonstrate that ostensibly unrelated proteins, such as Rap2 and agrin, play significant roles in ECM sensing and regulation of YAP/TAZ activity.

### 5.4. ECM-Mediated Tumour Initiation and Migration

A crucial hallmark of carcinoma and other cancer cells is their ability to migrate through surrounding tissues, penetrating the adjacent basement membrane. This dense, highly cross-linked membrane of ECM serves not only as an anchor for epithelial cells to surrounding connective tissue, but also as a significant barrier to epithelial cell migration [165]. However, due to the need for cells to migrate within the body during healthy tissue homeostasis, cancer cells have adopted a few methods of traversing the collagenous barrier [166]. One such method is the use of mechanical force. Mechanical force has increasingly been seen as a compelling factor in triggering the breaching of the basement membrane. As epithelial cancer cells proliferate, they are spatially constrained by the bordering basement membrane. This burgeoning population of cancer cells significantly increases the mechanical stress along the membrane, ultimately causing rupture and allowing cells to escape their microenvironment (Figure 3) [167].

Another method of membrane navigation is anchor cell invasion, in which anchor cells breach the basement membrane using protrusive, F-actin rich subcellular structures called invadopodia [168,169]. Indeed, electron micrographs of invasive tumours have demonstrated that leading invasive cells extend a single protrusive arm into the basement membrane [170]. After initial breach by the invadopodia, the membrane fissure widens, allowing for subsequent cells to traverse the collagen boundary [171]. However, along these breaching sites, elevated levels of collagen IV degrading products have also been found, indicating a possible third factor in the migration of cancer cells [172]. Increased accumulation of MMPs along the basement membrane has led to the commonly held assumption that proteases were solely responsible for degradation of the basement membrane. However, staining of the membrane during invasion reveal that laminin and collagen IV are in fact pushed aside by the invadopodia rather than fully degraded [165,167]. These results indicate that MMPs may, rather, play a role in the initial breaching of the basement membrane or in softening the matrix while anchor cell invadopodia facilitate direct invasion [166].

### 5.5. Metalloproteinases (MMPs) in Tumour Progression

The role of MMPs in cancer cell invasion is multi-pronged: they not only assist in the degradation of surrounding ECM barriers, but also release active growth factors and promote tumour angiogenesis [148]. The ECM is known to promote cell proliferation primarily through contact with the integrin family of cell surface receptors. However, certain ECM binding sites responsible for cell proliferation and survival have been shown to be “cryptic” or partially hidden within the ECM. MMPs simply unmask these hidden binding sites by degrading and loosening surrounding collagen, allowing for integrins along the cell membrane to interact directly with the matrix [148,173].

In addition to removing physical barriers and revealing cryptic binding sites, MMP-mediated degradation of collagen also exposes signalling components embedded within the ECM [173]. Stored in an inactive state when embedded within collagen, various growth factors are activated upon ECM degradation and allowed to bind with their target receptor. For example, MMP-2 mediated ECM degradation is known to release the active form of transforming growth factor-β (TGF-β). Upon its release, TGF-β is able to modulate cell invasion, immune response, and cell proliferation [174,175,176]. In effect, MMPs not only physically manipulate the surrounding ECM to allow for cell migration, but also create a microenvironment conducive to tumour development through growth factor release and cryptic binding site exposure. Thus, targeting MMPs could serve as a promising therapeutic approach, despite a previous lack of success (Box 2).

Despite MMP-induced angiogenesis, vasculature networks in the tumour are frequently disorganized with inter-capillary regions often exceeding the diffusion distance of oxygen. As such, hypoxia, or the state in which cells are devoid of oxygen, is a hallmark of cancer. In fact, measurements of the partial pressure of oxygen in tumours reveal that poorly oxygenated tumours strongly correlate to increased malignancy [173]. It is believed that cancer cells are able to withstand oxygen-derived regions by altering the transcription of various genes associated with angiogenesis. Hypoxia-inducible factors (HIF) are known play a crucial part in the regulating this intracellular cancer cell response to hypoxia [177,178,179]. Recent studies have indicated that HIF-1α, a member of the HIF family of transcription factors, has been associated with increased MMP and collagen production [177,178,179]. Importantly, HIF-1α is known to increase LOX deposition, ultimately stiffening the surrounding matrix [180]. Finally, HIF-1α has also been shown to activate transcription factors associated with epithelial–mesenchymal transition (EMT), the process by which cells lose their polarity and adhesion with adjacent cells, augmenting the invasive behaviour of cancer cells [180].

Box 2 **MMPs as a therapeutic target.**

As the role of the ECM in tumour progression becomes more apparent, cancer therapeutic interventions have begun to target key elements of the ECM in an attempt to limit metastasis. One key ECM component that has been targeted is the MMP family of enzymes. Due to the significant role of MMPs during cancer progression, the pharmaceutical industry has worked to develop safe therapies to inhibit MMP activity [181,182]. Several groups of synthetic MMP inhibitors, such as Marimastat, Minocycline, and Matimastat, have been developed to target broad groups of MMPs and tested in stage III clinical trials in late stage cancer patients [183].

Unfortunately, most therapies specifically targeting MMP activity demonstrated poor outcomes during clinical trials [184]. A few possible explanations exist for the poor clinical outcomes. Firstly, patients selected to receive the MMP-inhibiting therapies were late-stage cancer patients. As previously discussed, MMPs are known to play a role in tumour initiation and progression. It is possible that MMP-inhibiting agents could be more effective in early stage patients. Moreover, it is known that specific MMPs play different roles during cancer progression. It is likely that synthetic inhibitors need to be developed to target unique MMP subgroups at specific timeframes during cancer progression.

### 5.6. Role of Mechanical Stress in Tumour Growth and Treatment

As cancer progresses, tumours rapidly grow in size and stiffen due to the increased appearance of structural components, such as ECM, cancer-associated fibroblasts (CAFs), and cancer cells. This rapid rise in the rigidity of tumours is indeed one of the only easily detectable mechanical features of tumours that aid physicians in predicting malignancy and prognosis [185,186,187,188]. As the tumour progresses and stiffens, internally generated forces allow the tumour to disarrange adjacent healthy tissue and migrate into surrounding spaces. Accordingly, tumour progression is directly facilitated by these intratumour-generated forces and forces arising from interactions with its microenvironment [189]. These mechanical forces induce two unique types of stress on tumour cells: fluid and solid stress [190].

Generally, solid stress is created by the non-fluid components of the tumour. Initial evidence for the existence of solid stress within tumours came from the realization that blood and lymphatic vessels are mechanically compressed during tumour formation [191]. Growth-induced solid stress accumulates within tumours as the cancer cells rapidly proliferate. During this rapid reproduction process, cells grow into one another and strain the tumour microenvironment, which ultimately strains the surrounding healthy tissue [192]. In addition to intratumour-generated solid stress, externally generated solid stress accrues due to the adjacent tissue, which attempts to resist tumour expansion. In brief, solid stresses directly influence tumour progression in two manners: they first apply direct mechanical stress on cancer cells to alter genetic expression and, therefore, increase malignancy and invasion [193]. Secondly, solid stress deforms blood and lymphatic vessels to induce hypoxia [194].

As the name suggests, fluid stresses stem from forces generated by the fluid elements of the tumour. This includes shear stresses created by blood and lymphatic flow within the vessels, microvasculature (capillaries), and interstitial fluid flow [195,196]. In fact, fluid stress and solid stress are highly intertwined, as compression of blood/lymphatic vessels by solid stress greatly influences the fluid stress exerted on the surrounding epithelial tissue [190,197,198]. Vessel constriction reduces the cross-sectional area of the vessel to increase resistance to lymphatic flow, which in turn increases shear stress, interstitial fluid volume, and decreases perfusion rates [199]. This decrease of perfusion rates and flow significantly limits the ability for lymphatic vessels to remove excess fluid from the tumour, which ultimately increases interstitial fluid pressure in adjacent tumour tissue. Moreover, compression of blood and lymphatic vessels has significant negative ramifications for the effectiveness of chemo and immunotherapies [200].

Elevated solid and fluid stress within tumours place cancer cells in an entirely unique physiological environment. Increases in tension and compression acting on the cells mechanically activates tumourigenic pathways, increases proliferation rates, and promotes collective migration [146,148]. In addition to elevated rigidity, cancer cells produce and therefore are exposed to an elevated level of force than adjacent tissues [202]. While the bulk rigidity of tumours is relatively simple to quantify, measuring solid stress within tumours has proven to be a much more elusive task. Researchers have recently begun to quantify solid stress in individual tumour cells. Nia et al. [202] has recently provided the experimental framework for creating in-situ two-dimensional mapping of solid stress.

**Figure 4:**
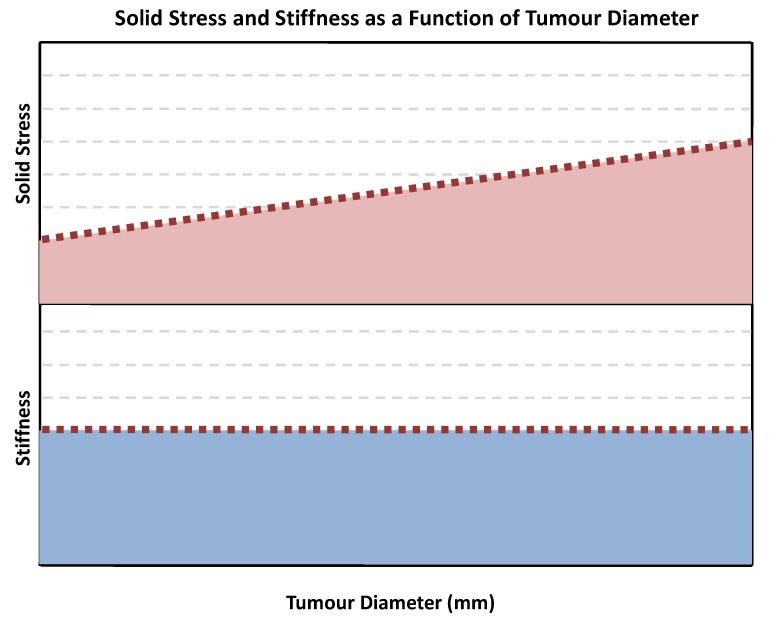
Solid stress and stiffness as a function of tumour diameter (adapted from Nia et al.) [201]. As rigidity of the ECM remains constant, an increase in tumour diameter is associated with increased solid stress within the tumour.

Researchers accomplish this mapping by releasing the solid stress within tissues in a controlled method using predefined geometry that encapsulates the tumour in agarose gel and records deformation after a precise incision is made. Combination of mathematical modelling and experimental analysis has revealed a few important findings: solid stress increases linearly with tumour size while rigidity remains constant, and adjacent healthy tissue contributes significantly to the solid stress within the tumour. These findings suggest that rigidity of the tumour is decoupled from the solid stress implemented on tumour cells (Figure 4).

### 5.7. Quantification of Tumour Cell Mechanical Stress in vivo

Tissue development, growth, and regeneration are crucially dependent on spatiotemporal variations in microenvironment mechanics. However, most current techniques for stress quantification utilize two-dimensional analysis that can only be performed in vitro, limiting its application for determining microenvironment mechanics during tumour progression. To address this issue, researchers in the Campàs group recently developed a novel oil micro droplet technique to quantify local cell-generated mechanical stresses in tumours in a spatiotemporal manner [201,203,204,205].

**Figure 5:**
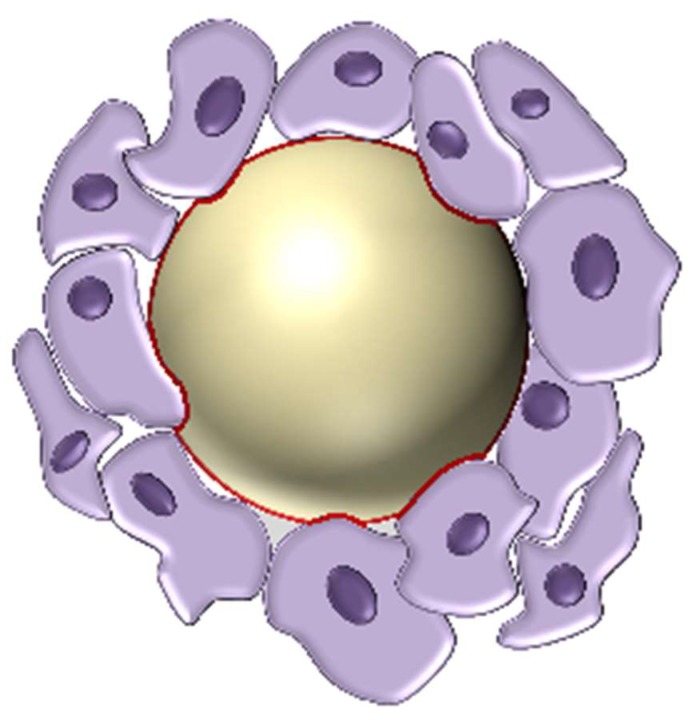
Schematic of oil micro droplet in vivo stress quantification described by Campàs et al. [203]. An oil droplet with calibrated surface tension is injected into living embryonic or cancerous tissue. As cells proliferate, they exert force onto the micro droplet and deform it. Deformations in curvature (red) of the oil droplet are used to calculate anisotropic stress within the tissue.

Fluorescent oil micro droplets with calibrated surface tensions are injected between tumour cells in living tissue while fluorescence microscopy is used to image localized oil droplet deformation. Provided droplet surface tension, measurements of curvature deformation along the oil droplet yield precise information regarding localized anisotropic mechanical stresses exerted by adjacent cells [201,203]. This technique of localized stress quantification shown above in Figure 5 revealed that the magnitude of cell-generated stress varies only weakly spatially during tumour progression, but increases dramatically over time [205]. Campàs et al. [203] further adapted the oil micro droplet technique to incorporate a biocompatible ferrofluid magnetic micro droplets to serve as mechanical actuators [206,207]. Using this technique, researchers are able to actively apply localized stress on tissues while observing tissue mechanical response. Indeed, this novel ferrofluid micro droplet allows for the simultaneous measurement of tissue mechanical properties and local cell-generated mechanical stress [207].

### 5.8. Role of ECM Mechanics in Behaviour of Myofibroblastic Cells

Many aggressive malignancies, such as pancreatic ductal adenocarcinoma (PDAC), are characterized by extensive desmoplasia and collagen deposition, which ultimately increases the rigidity of the tumour. Myofibroblast-like cells, such as pancreatic stellate cells (PSCs), are crucial mediators in the production of this fibrotic ECM [208]. When quiescent, PSCs are responsible for ECM turnover and remodelling through the production of MMPs. During wound repair, PSCs become activated by numerous soluble factors, including IL-1, IL-6, and TGF-β [141,142]. Alternatively, PSCs in the tumour desmoplasia of human pancreatic cancers behave erratically, become chronically activated, and create a microenvironment conducive to tumour growth [144]. It has been shown that pancreatic tumour cells are able to induce activation of PSCs through increased secretion of TGF-β1 and PDGF [141]. However, recent studies have indicated that PSCs may be able to sustain activation due to the mechanical properties of the microenvironment alone [209,210].

Our recent work has shown that matrix stiffness is sufficient for activation of PSCs. Upon activation, PSCs were found to mechanically sense the increased rigidity of the environment as they produce excess collagen [210,211]. This mechanosensing of tissue stiffness activates intracellular-signalling pathways within the PSC, encouraging the myofibroblast-like cell to produce excess deposits collagen. This process of stiffness mechanosensing forms a positive-feedback loop, in which PSCs continue to secrete collagen as the matrix becomes stiffer and stiffer [210,212]. Moreover, we also found that matrix rigidity influences PSC migration, as PSCs migrate from adjacent soft tissue towards the stiff tumour microenvironment [212]. Thus, as the matrix is stiffened, distant PSCs are recruited towards the stiff tissue and become activated, further enhancing the positive feedback loop.

Inactivating the mechanosensing and remodelling capability of PSCs may be an effective therapeutic strategy. All-trans-retinoic acid (ATRA) has been shown to suppress PSC mechanosensing by downregulating MLC-2 actomyosin contractility. This leads to PSC inactivation and turns off the positive feedback loop of increased matrix rigidity and PSC activation [213]. By inactivating PSC’s ability to sense the mechanical environment, ATRA reduces fibrosis and suppresses cancer invasion. Furthermore, inactivation of PSC’s ECM remodelling capability prevents its ability to mechanically liberate TGFβ from Latent TGFβ Binding Protein (LTBP) [214]. 

These results indicate that the mechanical environment is a powerful regulator of PDAC progression via PSC activation and ECM remodelling, suggesting that reprogramming of PSCs and other resident cells may be a viable therapeutic target to alleviate tumour growth. A review of the role of mechanical rigidity and mechanical stress in tumour proliferation can be seen in Box 3.

Box 3 **Rigidity and stress influence cell behaviour.**

As discussed in the previous three sections, mechanical stiffness and stress of the tumour both play extremely important yet distinct roles in influencing cancer cell behaviour. Although there is significant overlap in effects of rigidity and mechanical stress, the points below highlight certain differences:

**Tumour Rigidity**
  ❖ Caused by elevated ECM deposition
  ❖ Mechanically activates pancreatic stellate cells (PSCs) to produce ECM
  ❖ Induces EMT in epithelial cells
  ❖ Amplifies growth-factor signalling
**Tumour Stress (Solid and Fluid)**
  ❖ Caused by increased cell proliferation and blood flow
  ❖ Induces hypoxia in tumours
  ❖ Augments cell proliferation
  ❖ Increases chemo resistance

### 5.9. Seed and Soil

For many years, the scope of study regarding cancer metastasis had primarily focused on cancer cells and their migration through the vasculature. However, as the complex interplay between the ECM and cancer progression became apparent, the perspective on cancer progression to distant organs evolved. In 1889, surgeon Stephen Paget posited that cancer metastasis is dependent on complex interactions between the migrating tumour cells (the “seed”) and its microenvironment (the “soil”) [208,209]. Despite doubt over the course of the twentieth century, Paget’s hypothesis was strengthened in the 1970s when Isaiah Fidler’s research demonstrated that successful tumour migration could only occur at certain organ sites [215].

Further research demonstrated that primary tumours have the ability to induce the formation of a microenvironment at distant organ sites that are conducive to tumour growth [209,213]. This newly formed distant microenvironment, known as the pre-metastatic niche (PMN), promotes tumour growth through a variety of methods, including increased inflammation, vascular permeability, immunosuppression, and tissue stiffness [215,216,217]. Studies have indicated a variety of molecules and mechanisms involved with the generation of the PMN, such as tumour-derived secreted factors (TDSFs) and tumour-derived extracellular vesicles (EVs) [218,219].

**Figure 6:**
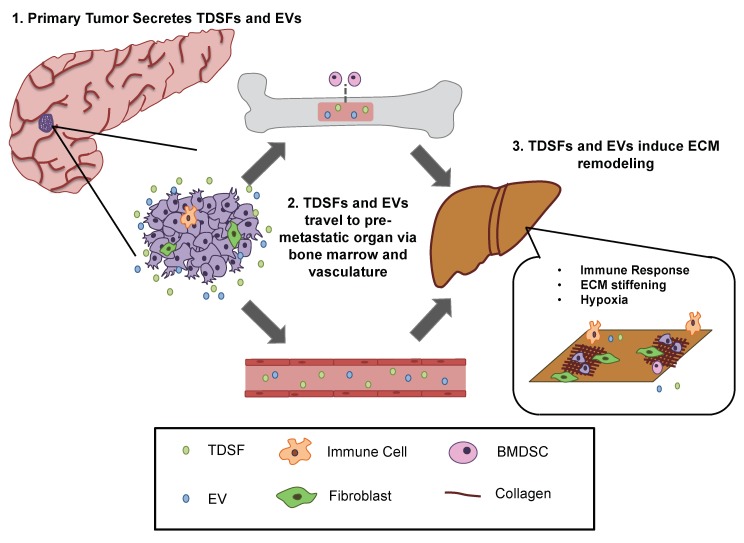
Illustration of pre-metastatic niche formation in the liver. (**1**) The primary tumour located in the pancreas emits tumour-derived secreted factors (TDSFs) and extracellular vesicles (EVs). (**2**) TDSFs and EVs migrate through the vasculature and bone marrow to the secondary organ. While in the bone marrow, TDSFs and EVs recruit bone marrow-derived stem cells (BDSCs), such as hematopoietic stem cells, to the secondary organ site. (**3**) TDSFs and EVs induce immune cell recruitment and ECM remodelling through LOX and cancer-associated fibroblasts at the pre-metastatic site. [220]

One primary form of tumour-derived EV is the exosome, which has a diameter ranging from 30–100 nm [218]. Exosomes containing proteins, mRNAs, and unique ECM-binding integrins are first secreted from the primary tumour and travel through the vasculature, promoting vessel leakiness in distant organ sites. EVs and TDSFs, such as TGF-β and MMP-9, then alter local resident cells and fibroblasts [219,221]. Fibroblasts altered by exosomes recruit more inflammatory cytokines, such as TGF-β, and dramatically increase collagen deposition to stiffen tissue in the PMN [222]. Just as in primary tumour tissue, elevated collagen deposition and inflammation increase interstitial stress within the tissue to induce hypoxia [220]. Finally, TDSF-mediated recruitment of non-resident cells, such as bone marrow-derived cells (BMDCs) and immune suppressor cells, ultimately attracts circulating tumour cells (CTCs) to the site (Figure 6) [223]. CTCs traveling through the vasculature are able to easily permeate into the PMN due elevated vasculature leakiness and collectively migrate through the organ toward the stiffer tissue, in a process known as durotaxis [224,225].

Interestingly, the notion of the PMN proposes that critical changes to the ECM, typically associated with primary tumour formation, can occur prior to the arrival of tumour cells [226,227]. The Seed and Soil process closely mirrors the process of primary tumour ECM remodelling, with one critical difference: it occurs in the absence of cancer cells. Furthermore, it specifies that TDSF and EV-mediated ECM remodelling is crucial in facilitating cancer metastasis. However, many aspects of the mechanism regarding PNM formation remain elusive.

Although exosomes have been known to play a crucial role in PMN development, why cancer cells only metastasize to specific organs, a process known as organotropism, has remained unclear. Recent research in the Lyden laboratory has demonstrated that exosomes secreted by primary tumour cells have specific integrin expression patterns that dictate organotropism. Indeed, exosome proteomics revealed that exosomal integrins α6β4 and α6β1 are closely linked with lung metastasis, while exosomal integrin αvβ5 is associated with liver metastasis [220]. Targeting of these specific integrins decreased exosomal uptake by resident cells and decreased lung and liver metastasis, respectively. These results suggest that exosomal integrin expression could be used to predict organ-specific metastatic sites. Moreover, there is an implication that cancer therapies may be most beneficial if tailored to distinct metastatic sites (lung, liver, etc.) and each stages of cancer metastasis: pre-metastatic and post-metastatic [215].

## 6. Challenges and Future Perspectives

In this review we have discussed the complex and nuanced role of the ECM in tissue development and cancer progression. Over the past 20 years, studies have revealed the importance of the ECM in regulating crucial physiological processes such as stem cell lineage specification, cell migration, and proliferation [138,139,140]. Accordingly, perspectives have shifted to address cancer not only as a disease of uncontrolled cell proliferation, but also of dysregulation of the microenvironment. The ostensibly static ECM actively undergoes dynamic remodelling during all stages of cancer progression in a complex interplay between cancer cells, resident cells, and non-cellular components. Advances in understanding of the role of ECM in cancer progression have provided hope and revealed promising therapeutic targets for mitigating cancer’s ability to metastasize. However, a lack of success in targeting broad ranges of proteins, such as MMPs and collagen, reveals the temporal sensitivity and specificity needed to effectively limit the spread of the tumour cells.

As neoplastic cells proliferate rapidly in tumours, they experience an increase in mechanical stress, which mechanically activates tumourigenic pathways, increases migration, and induces hypoxia. An essential region of future cancer research will be to ascertain the mechanism by which increased mechanical stress in tumours relates to malignant behaviour and angiogenesis. These signalling pathways relating to exogenous mechanical stress and malignant behaviour serve as an auspicious therapeutic target to resist cancer progression. Moreover, understanding the relationship between increased solid stress and angiogenesis mechanisms would elucidate possible advancements for drug delivery. Recent developments in in vivo cell stress quantification techniques may provide novel insights into the relationship between ECM-generated stress and hypoxia [213,214,215,216].
